# Factors Associated with Singleton Preterm Birth in Shire Suhul General Hospital, Northern Ethiopia, 2018

**DOI:** 10.1155/2019/4629101

**Published:** 2019-05-08

**Authors:** Bayew Kelkay, Awol Omer, Yelfu Teferi, Yohannes Moges

**Affiliations:** ^1^School of Midwifery, College of Medicine and Health Science, University of Gondar, Gondar, Ethiopia; ^2^Department of Midwifery, College of Health Science and Comprehensive Specialized Hospital, Aksum University, Axum, Ethiopia; ^3^Department of Midwifery, Institute of Medicine and Health Science, Debre Berhan University, Debre Berhan, Ethiopia

## Abstract

**Background:**

Preterm birth is the leading cause of neonatal mortality and significant health consequences to the newborn, families, and communities and tens of emotional and economic costs. Therefore, the aim of this study was to assess the magnitude of singleton preterm birth and associated factors in Shire Suhul General Hospital, Northern Ethiopia.

**Methods:**

Institutional based cross-sectional study was conducted among systematically selected 325 postnatal mothers in Shire Suhul General Hospital, Northern Ethiopia, from January to March 2018. The data were collected through both face-to-face interview and chart review by using pretested semistructured questionnaires. SPSS version 20 was employed to enter and analyze the data. Both bivariate and multivariate logistic regression models were run to identify factors associated with singleton preterm birth at the level of* P* values ≤ 0.25 and <0.05 for each model, respectively.

**Result:**

The magnitude of singleton preterm birth in Suhul Hospital was 16.9%. Smoking cigarette/drinking alcohol during pregnancy [AOR=3.61: CI 95%; 1.59-8.23], previous abortion [AOR=2.37: CI 95%; 1.15-4.88], hemoglobin level < 11gm/dl [AOR=2.44: CI 95%; 1.14-5.22], visible physical neonatal congenital anomaly [AOR=10.42: CI 95%; 1.66-65.23] , and history of giving low birth weight baby [AOR: 2.78 CI 95%; 1.39-5.55] were the factors statistically associated with singleton preterm birth.

**Conclusion:**

The magnitude of preterm birth in this study was higher than the average prevalence of preterm birth reported in Ethiopia. Smoking cigarette/drinking alcohol during pregnancy, mothers who had previous abortion, low maternal hemoglobin level, presence of visible physical congenital anomalies of newborn baby, and history of bearing low birth weight baby were found to have statistically significant association with singleton preterm birth. Supplement of daily iron with folic acid (folic acid ideally before pregnancy) for all pregnant mothers with good adherence monitoring and giving attention to preconceptional health care service to avoid any cigarette smoking/alcohol drinking and risk detection were set as recommendations.

## 1. Introduction

The World Health Organization (WHO) defines preterm birth as a baby born too early or before 37 complete weeks of gestation from the first day of the last normal menstrual period (LNMP). Birth too soon is categorized as extremely preterm (less than 28 weeks), very preterm (28-32 weeks), and moderate to late preterm (32weeks to 36 weeks and 6 days). Preterm birth may happen spontaneously or is initiated by clinicians for different medical or nonmedical reasons and mothers deliver either single or multiple neonates. Globally, around 1 million children die each year due to complications of preterm birth and 60% of them occur in Africa and South Asia. Generally, the average rate of preterm birth in lower income countries and higher income countries is 12% and 9%, respectively [1]. In Ethiopia, 320,000 premature babies are born each year and it directly contributes to 24,400 (12.5%) deaths of under-five children [2].

Preterm birth is the major single burden for both developing and developed countries. Some of the anticipated complications following birth of preterm neonates are motor development, behavior and academic performance impairment, and even deaths within minutes [3, 4]. Preterm birth often requires more resources for health care, social based rehabilitation service, special education placement, and specialized health care professionals throughout their childhood and even adulthood [5].

According to pieces of literature, several sociodemographic and fetomaternal factors have contributed to the overall rise in the prevalence of preterm birth. Experience of previous preterm delivery [6–9], mothers having inadequate prenatal visits [10–12], pregnancy that induced hypertension during pregnancy [10, 13, 14], anemic mothers [15, 16], antepartum hemorrhage during the most recent pregnancy [8–10, 17], twin delivery in most recent birth [10], presence of fetal anomaly [18, 19], smoking [20, 21], and HIV positive mothers [14] were some of repeatedly associated factors with the occurrence of preterm birth.

A high epidemic of preterm birth and related newborn deaths is one of the greatest health challenges of these days. However, two-thirds of these complications and deaths could be prevented by simple inexpensive measures that do not need high technology [22, 23]. In developed countries, treatment with metronidazole and erythromycin helps to prevent premature delivery caused by bacterial vaginosis [24]. Using vaginal progesterone is the most efficacious and safe method to prevent preterm labor that leads to preterm birth [25].

However, preterm birth is a complex multifactorial process associated with diverse pathogenetic mechanisms. Most factors are usually prevented by simple strategies. Health care providers or other stakeholders who worked in this study setting need data related to common regional factors, since data limitation is there. Few studies conducted in Ethiopia are related to preterm birth. These studies were conducted by direct mothers' interview, and in the meantime, researchers have missed very important variables that need to be examined but valid data were accessible from maternal charts like maternal hemoglobin level, neonatal weight, gestational age determined by clinicians (using ultrasound or LNMP), and diagnosed medical diseases. Additionally, almost all studies included mothers who gave multiple (twin, triple, and higher) and singleton fetus indifferently. Meanwhile, multifetal pregnancies do not have an equal chance suffering for preterm birth compared to singleton birth due to physiological reasons. Therefore, answering the above issues and using it as input for the study area to conduct further studies were the aim of this study.

## 2. Materials and Methods

### 2.1. Study Design and Population

Institutional based cross-sectional study was conducted from January to March 2018 with 325 mothers who gave birth in Shire Suhul General Hospital at the time of data collection. The study was conducted at Shire town, the capital of northwest zone of Tigray regional state located 1087Kms far from Addis Ababa (the capital city of Ethiopia) and 357 Kms from Mekelle (the capital of Tigray regional state). Based on the reports from the zonal administrative office, the town has 67,243 total populations; among these, 36,146 are females. In Suhul General Hospital, the total annual birth report in 2017/18 was 3,048 and the first quarter (July-October) birth was 720.

### 2.2. Inclusion and Exclusion Criteria

Mothers who gave birth and had known either LNMP or 1st trimester ultrasound diagnosis were included for this study.

### 2.3. Sample Size and Sampling Technique

Sample size for this study was calculated by using single population proportion formula with the assumption of 95% confidence level, 25.9% prevalence of preterm birth [19], and 5% marginal error. The final sample size after considering 10% of nonresponse rate was 325. Finally, a systematic random sampling technique with sampling interval (K^th^) value of two (2) was used to identify interviewed participants. The first study participant was selected randomly and continued every two intervals.

### 2.4. Study Variables

#### 2.4.1. Dependent Variable

Singleton preterm birth refers to a mother who gave one baby with gestational age < 37 weeks or before 259 complete days.

#### 2.4.2. Independent Variables

*Sociodemographic variables* are age, marital status, religion, residence, occupation, and educational status.* Maternal factors (obstetrical & medical ) *are parity, gestational age of current baby in weeks, medication intake during the most recent pregnancy, smoking cigarette/drinking alcohol during the most recent pregnancy, history of hyperemesis-gravidarum in the most recent pregnancy, previous abortion, previous stillbirth, previous preterm birth, previous cesarean delivery, history of vaginal bleeding during the most recent pregnancy (antepartum hemorrhage), history of diagnosed urinary tract infection in the most recent pregnancy, hypertension disorder during the most recent pregnancy, history of hospitalization during the most recent pregnancy, previous diagnosed fibroid, maternal current serostatus, level of the most recent hemoglobin, previous malaria attack, mode of delivery of the current baby, history of ANC (antenatal care) follow-up, number of ANC follow-ups, and birth (interdelivery) interval.* Fetal factors* are congenital abnormalities of the current newborn, previous multiple delivery, giving congenitally defected baby, and history of giving LBW (low birth weight) baby including the most recent and sex of the current newborn.

### 2.5. Data Collection and Quality Control

The data were collected by using a pretested semistructured questionnaire through both face-to-face interview and extracting (review) respective mothers' chart for necessary data. Questionnaires were prepared first in English after reviewing different works of literatures and translated into local language (Tigrigna) and then back to English by the third party who was native in Tigrigna to ensure consistency. The data collection was carried out within 24 hours before mothers going to be discharged. Four midwives who were fluent in local language were involved in data collection. Detail orientation was provided for data collectors.

### 2.6. Data Processing and Analysis

Collected data were entered and cleaned and analysis was by SPSS version 20. Descriptive statistics were summarized into frequency and proportion. Both bivariate and multivariate analysis model were run to check the association between dependent and independent variables. All variables with* P* value ≤ 0.25 in bivariate analysis were taken into multivariate logistic regression model and variables having* P* value < 0.05 with 95% CI declared factors significant association with singleton preterm birth.

## 3. Result

### 3.1. Sociodemographic Characteristics

A total of 325 mothers participated in this study with 100% response rate. Two hundred seventy-three (84%) of the participants were of age <35 years with the mean age of 27.88 years±5 years of SD. More than three-fourths (88.6%) of the study participants were Orthodox Christian followers and majority, 306(94.2%), were married.

More than half (57.8%) of the mothers were from the urban area and 201 (61.8%) of them were the housewives in occupational status. More than three-fourths, 252 (77.5%), were primary school and less in their educational status. Family size of more than half, 189 (58.2%), of the mothers was within the range of 2-4 individuals. Almost all, 304 (93.5%), mothers have never suffered from hard workload in the most recent pregnancy and 91 (28%) participants' monthly income was within the range of 1401-2350 Ethiopian Birr ( see Table 1).

### 3.2. Maternal (Obstetrical and Medical) Factors

**Figure 1:**
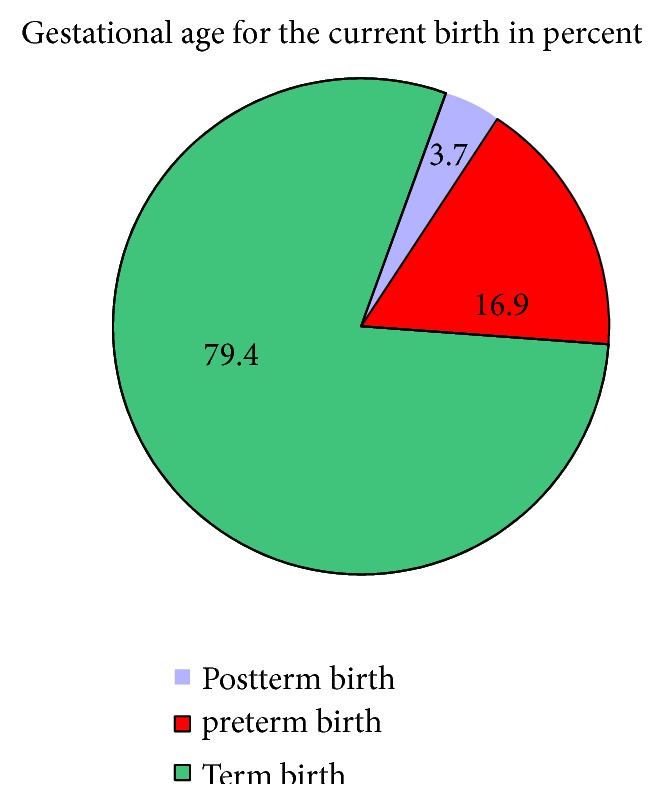
Gestational age of newly born neonates in Shire Suhul General Hospital, Northwest Tigray, Ethiopia, 2018 (N=325).

According to this study, greater than three-fourths of the study participants gave term neonates followed by 16.9% of preterm birth (see Figure 1).

Nearly half, 167(51.4%), gave within the range of 2-4 viable babies (multipara) before the study period. More than 80% of the study participants have never had the history of medication intake, drinking alcohol/smoking cigarette, vaginal bleeding after fetal viability (APH), urinary tract infection, hypertension, hospital admission, and hyperemesis-gravidarum in most recent pregnancy. Two hundred sixty-eight (82.5%), 284 (87.4%), 282 (86.8%), 295 (90.8%), and 310 (95.4%) had no history of abortion, stillbirth, preterm birth, cesarean delivery, and fibroids (uterine mass) in previous pregnancies.

Among the study participants, 16 (4.9%) were reactive in HIV/AIDS, 91 (24.9%) were attacked by malaria, and 48 (14.8%) mothers' hemoglobin levels were <11gm/dl. Of 325 study participants, 256 (78.8%) mothers gave birth vaginally and most, 134 (41.2%), of them had average interval of birth between 1 and 3 years. Almost all, 302 (92.9%), of the study participants had the history of ANC follow-up during the recent pregnancy and from those majority, 245(81.1%), had ≥4 contacts (see Table 2).

### 3.3. Fetal Factor

Among newborns who were delivered during the study period, only 6 (1.8%) mothers gave congenital abnormal babies whereas 17 (5.2%) of the respondents had a previous history of giving congenital anomalies baby. Including the most recent births, 60 (18.5%) of the study participants had the history of having low birth weight baby. More than half, 183 (56.3%), neonates were female (see Table 3).

### 3.4. Factors Associated with Singleton Preterm Birth

All independent variables were analyzed using binary logistic regressions and all variables with* P* value ≤ 0.25 were fitted to run in multivariate logistic regression. Finally, in multivariate logistic regression model, smoking cigarette/drinking alcohol during the most recent pregnancy, history of abortion, hemoglobin level, a physical congenital defect in the most recent baby, and history of bearing low birth weight baby including the most recent one were identified as independently associated variables with singleton preterm birth at* P* value < 0.05.

Mothers who smoked cigarette/drank alcohol during the most recent pregnancy were 3.6 times more likely to have a preterm delivery than those who did not smoke/drink [AOR=3.6; 95%CI: 1.59-8.23]. Similarly, mothers who had a history of abortion were nearly 2.4 times more likely to give preterm baby than their counterparts [AOR=2.37; 95%CI: 1.15-4.88].

Mothers whose most recent hemoglobin level was <11 gm/dl were 2.4 times more risky for preterm birth as compared to those whose hemoglobin level was ≥ 11gm/dl [AOR=2.44; 95%CI: 1.14-5.22]. Finally mothers who gave neonates with physical congenital defect and who had history of delivering low birth weight baby including the most recent birth were statistically significant with singleton premature birth compared to their counterparts [AOR=10.4; 95%CI: 1.66-65.2] and [ AOR=2.78; 95%CI: 1.39-5.55], respectively ( see Table 4).

## 4. Discussions

This study revealed that the prevalence of preterm birth was 16.9%. This finding is in line with those studies conducted in India 18.01% [26], Nigeria 16.8% [27], Malawi 16.3% [16], Tanzania 14.2% [12], and Kenya 18.3% [9]. This similarity could be due to some related leveled socioeconomic status and lifestyle among participants since all are developing countries.

This finding is higher than the average rate of preterm birth in lower income countries reported by WHO 12% [28], Brazil 12.3% [29], Iran 5.1% [7], Australia 6.8% [21], Debre Markos 11.6% [30], Gondar 4.4% [14], average Ethiopian prematurity birth 10% [23], and Tigray 8.1% [31]. This disparity could be due to this study setting having low access to quality of health care this study carried being out in single hospital and small sample participants involved in this study. However, most studies conducted in the above regions were case-control, involved multiple institutions, and used a large sample size.

On the other side, the magnitude of preterm birth in this study was much lower than the study carried out in India 25.6% [32], Indonesia 35% [15], and Jimma University Specialized Hospital 25.9% [19]. This variation might be due to the fact that multiple pregnancies were not included in this study, nature of study areas, and health-seeking behavior among the study participants.

Mothers who smoked cigarette/drank alcohol during the most recent pregnancy experienced preterm birth more than those who never smoked/drank [AOR=3.61; 95%CI: 1.59-8.23]. This finding was consistent with that of the study conducted in China, Australia, and Jimma [19–21]. This could be explained by the fact that cigarette/alcohol may interfere with the nutritional process and affect the strength of the membrane negatively that leads to premature rupture of the membrane. Mothers who had previous history of abortion were also 2.4 times more likely to give preterm baby [AOR=2.37; 95%CI: 1.15-4.88] than those who never had. This is in agreement with the study conducted in Iran, Tanzania, and Jimma [12, 17, 19]. This finding could be explained by the fact that infection following the procedure may result in intra-amniotic infection and possibly idiopathic risk factor for preterm premature rupture of membrane and then result in preterm labor.

Mothers whose most recent hemoglobin levels <11gm/dl were nearly 2.5 times more likely to experience preterm birth compared to those ≥11gm/dl [AOR=2.44; 95%CI: 1.14-5.22]. This figure is in line with WHO 2013 report and the studies carried out in India, Indonesia, Malawi, and Debre Markos [13, 15, 16, 30, 32]. This could be explained biologically, anemia (<11gm/dl) cause for hypoxia which can induce maternal and fetal stress, which stimulate the production of the corticotrophin-releasing hormone to lead the initiation of preterm labor.

Visible physical neonatal congenital anomalies in the most recent baby increased odds of the preterm birth occurrence compared to normal babies with [AOR=10.4; 95%CI: 1.66-65.2]. This finding was consistent with that of the study conducted in the United States of America, Palestine, and Jimma [11, 18, 19]. This could be because of clinicians who intended to initiate the labor artificially as soon as they detected congenital anomalies fetus who will not be compatible in life irrespective of gestational age. Mothers who had the previous history (including the most recent) of bearing neonatal weight <2500gm were 2.8-fold more likely to give preterm baby compared to their counterparts. This finding was similar to the study carried out in Tanzania [12]. The possible reason related to indirect relations was because that problems with the placenta and maternal infection which could prevent oxygen and nutrients to reach the fetus result in both preterm and low birth weight baby.

## 5. Limitation of the Study

The study was conducted with a small sample size, only in one institution, and used maternal charts as additional data source which could result in overreporting and difficulty in inferring the entire society. Additionally, we failed to assess hypertension disorders during pregnancy separately as preeclampsia, eclampsia, gestational hypertension, and superimposed preeclampsia. Finally, the study design was cross-sectional, which did not show the causality of the limitations of this study.

## 6. Conclusion

The magnitude of preterm birth in this study was higher than the average prevalence of preterm birth reported in Ethiopia. Substance intake during pregnancy, previous abortion, low maternal hemoglobin level, the presence of visible physical congenital anomalies of newborn baby, and history of bearing low birth weight baby had statistically significant association with preterm birth. Supplement of daily iron with folic acid (folic acid ideally before pregnancy) for all pregnant mothers with good adherence monitoring and giving attention to preconceptional health care service to avoid cigarette/alcohol intake, and risk detection were set as recommendations.

## Figures and Tables

**Table 1:** Sociodemographic characteristics of respondents in Shire Suhul General Hospital, Northwest Tigray, Ethiopia, 2018 (n=325).

Variables	Variable category	Frequency	Percent (%)
Age in year	< 35	273	84
	≥ 35	52	16
Marital status	Single	13	4
	Married	306	94.2
	Widowed	4	1.2
	Divorced	2	0.6
Religion	Orthodox	288	88.6
	Muslim	35	10.8
	Protestant	2	0.6
Residence	Rural	137	42.2
	Urban	188	57.8
Occupation	Student	15	4.6
	Merchants	64	19.7
	Housewife	201	61.8
	Governmental workers	47	14.5
Educational status	Primary or less	252	77.5
	Secondary education	10	3.1
	Postsecondary education	63	19.4
Family size	2-4	189	58.2
	>=5	136	41.8
Suffering from a heavy workload	Yes	21	6.5
	No	304	93.5
Monthly income of the family in birr	150-650	50	15.4
	651-1400	74	22.8
	1401-2350	91	28
	2351-3550	65	20
	3551-5000	29	8.9
	>5000	16	4.9

**Table 2:** Obstetrical and medical characteristics of the respondents in Shire Suhul General Hospital, Northwest Tigray, Ethiopia, 2018 (n=325).

Variables	variable category	Frequency	Percent
Parity	Para 1	103	31.7
	Para 2-4	167	51.4
	Para ≥ 5	55	16.9
GA of the most recent pregnancy	<37 weeks	55	16.9
	37-41^+6^wks	258	79.4
	≥ 42 weeks	12	3.7
History of medication intake during the most recent pregnancy	Yes	57	17.5
	No	268	82.5
History of smoking cigarette/drinking alcohol during the most recent pregnancy	Yes	33	10.2
	No	292	89.8
History of hyperemesis gravidarum in the most recent pregnancy	Yes	64	19.7
	No	261	80.3
History of abortion	Yes	57	17.5
	No	268	82.5
History of stillbirth	Yes	41	12.6
	No	284	87.4
History of preterm birth in previous pregnancies	Yes	43	13.2
	No	282	86.8
History of Cesarean delivery	Yes	30	9.2
	No	295	90.8
History of vaginal bleeding during the most recent pregnancy(Ante-partum hemorrhage)	Yes	30	9.2
	No	295	86.8
History of diagnosed urinary tract infection in most recent pregnancy	Yes	17	5.2
	No	308	94.8
History of hypertension during the most recent pregnancy	Yes	20	6.2
	No	305	93.8
Hospitalization during the most recent pregnancy	Yes	29	8.9
	No	296	91.1
History of diagnosed fibroid(uterine mass)	Yes	15	4.6
	No	310	95.4
Maternal serostatus (HIV/AIDS)	Positive	16	4.9
	Negative	309	95.1
The most recent maternal Hgb level in mg/dl	<11mg/dl	48	14.8
	>=11mg/dl	277	85.2
History of malaria attack	Yes	91	24.9
	No	244	75.1
Mode of delivery for the most recent birth	Vaginal	256	78.8
	Cesarean	69	21.2
Birth interval in years	1-3	134	41.2
	>3	120	36.9
	First baby	71	21.8
ANC follow-up for the most recent Pregnancy	Yes	302	92.9
	No	23	7.1
If ‘Yes' number of visits(n=302)	<4 visits	57	18.9
	>=4 visits	245	81.1

**∗** ANC; Antenatal care, Hgb; Hemoglobin, GA; Gestational age and HIV; Human immune deficiency virus.

*∗*Medication/drug intake (any amount and type) when she was pregnant in that baby.

**Table 3:** Fetal characteristics of the respondents in Shire Suhul General Hospital, Northwest Tigray, Ethiopia, 2018 (n=325).

Variables	variable category	Frequency	Percent
Congenital abnormality of current newborn	Yes	6	1.8
	No	319	98.2
Previous multiple deliveries	Yes	30	9.2
	No	295	90.2
Previous history of congenital abnormality	Yes	17	5.2
	No	308	94.8
History of LBW including current newborn	Yes	60	18.5
	No	265	81.5
Sex of newborn	Male	142	43.7
	Female	183	56.3

*∗*LBW; Low birth weight

**Table 4:** Bivariate and multivariate factors associated with singleton preterm birth in Shire Suhul General Hospital, Northwest Tigray, Ethiopia, 2018 (n=325).

Variables(n=325)		Preterm birth		COR(95%CI)	AOR (95% CI)
		No	Yes		
Parity	P ≥5	39(70.9%)	16(29.1%)	1.00	-
	P 2-4	145(86.8%)	22(13.2%)		
	Primipara	86(83.5%)	17(16.5%)	0.48 [0.18-0.77]	0.78 [0.32-1.91]
Medication intake	No	230(85.8%)	38(14.2%)	1.00	-
	Yes	40(70.2%)	17(29.8%)	2.57 [1.33-4.99]	0.8 [0.32-1.99]
Smoked cigarette/drank alcohol	No	251(86.0%)	41(14 %)	1.00	-
	Yes	19(57.6%)	14(42.4%)	4.51 [2.10-9.70]	*3.61*[*1.59-8.23*]*∗∗*
Hyper-emesis- gravidarum	No	225(86.2%)	36(13.8%)	1.00	-
	Yes	45(70.3%)	19(29.7%)	2.64[1.39-5.011]	1.08[0.44-2.66]
History of abortion	No	233(86.9%)	35(13.1%)	1.00	-
	Yes	37(64.9%)	20(35.1%)	3.61[1.88-6.89]	*2.37*[*1.15-4.88*]*∗∗*
History of stillbirth	No	244(85.9%)	40(14.1%)	1.00	
	Yes	26(63.4%)	15(36.6%)	3.52[1.72-7.22]	0.85[0.28-2.65]
History of preterm	No	244(86.5%)	38(13.5%)	1.00	
	Yes	26(60.5%)	17(39.5%)	4.2[2.084-8.45]	1.64[0.67-3.99]
History of Vaginal bleeding	No	253(85.8%)	42(14.2%)	1.00	
	Yes	17(56.7%)	17(43.3%)	4.61[2.09-10.18]	1.51[0.50-4.55]
Hospital admission	No	251(84.8%)	45(15.2%)	1.00	
	Yes	19(65.5%)	10(34.5%)	2.94[1.28-6.73]	1.06[0.35-3.25]
Hemoglobin level before birth	≥11gm/dl	238(85.9%)	39(14.1%)	1.00	
	<11gm/dl	32(66.7%)	16(33.3%)	3.05[1.53-6.08]	*2.44*[*1.14-5.22*]*∗∗*
History of Malaria attack	No	211(86.5%)	33(13.5%)	1.00	
	Yes	59(72.8%)	22(27.2%)	2.38[1.29-4.40]	1.68[0.85-3.34]
Number of ANC visits (N=302)	≥ 4 visits	229(86.7%)	35(13.3%)	1.00	
	<4 visits	41(67.2%)	20(32.8%)	3.19[1.68-6.07]	1.43[0.64-3.20]
Visible physical Congenital defect	No	268(84.0%)	51(16.0%)	1.00	
	Yes	2(33.3%)	4(66.7%)	10.51[1.88-58.90]	*10.42*[*1.66-65.23*]*∗∗*
LBW	No	231(87.2%)	34(12.8%)		
	Yes	39(65.0%)	21(35.0%)	3.66[1.93-6.95]	*2.78*[*1.39-5.55*]*∗∗*

*∗*ANC: Antenatal care, P: Parity and LBW: Low Birth Weight

*∗∗* P value < 0.05 and significantly associated.

## Data Availability

The data used to support the findings of this study are available from the corresponding author upon request.

## Abbreviation

AOR: Adjusted odds ratio
ANC: Antenatal care
COR: Crude odds ratio
LNMP: Last normal menstrual period
SPSS: Statistical package for social sciences
WHO: World Health Organization.
